# Optimizing Hearing Outcomes in Nasopharyngeal Cancer Survivors in the Era of Modern Radiotherapy and Systemic Therapy

**DOI:** 10.3390/cancers16183237

**Published:** 2024-09-23

**Authors:** Jason C. S. Ho, Brigette B. Y. Ma, James C. H. Chow

**Affiliations:** 1 Department of Clinical Oncology, Queen Elizabeth Hospital, Hong Kong SAR, China; hcs472@ha.org.hk; 2 Department of Clinical Oncology, The Chinese University of Hong Kong, Hong Kong SAR, China

**Keywords:** nasopharyngeal carcinoma, radiotherapy, ototoxicity, hearing loss, sensorineural, survivorship

## Abstract

**Simple Summary:**

Sensorineural hearing loss is a common late complication among nasopharyngeal cancer survivors, primarily due to the close proximity of the auditory apparatus to the treatment volume and the use of cisplatin-based chemotherapy. The incidence of hearing loss in the era of intensity-modulated radiation therapy varies widely, influenced by factors such as patient demographics, auditory assessment methods, and the duration of follow-up. While guidelines have provided recommendations on radiation dose constraints to the cochlea to mitigate hearing loss, significant risks remain. To improve hearing outcomes, strategies such as radiotherapy de-escalation, personalized treatment planning, and the consideration of alternative systemic agents in localized nasopharyngeal cancer are being investigated. This review discusses the context and relevant evidence regarding potential strategies to improve hearing outcomes in this patient population.

**Abstract:**

Intensity-modulated radiation therapy (IMRT) improves disease control and reduces treatment-related toxicity in patients with localized nasopharyngeal carcinoma (NPC). However, due to the proximity of the auditory apparatus to the treatment volume and the frequent incorporation of cisplatin-based chemotherapy, treatment-related sensorineural hearing loss (SNHL) remains a common debilitating complication among NPC survivors. The reported crude incidence of SNHL following IMRT for NPC varies widely at 1–46% due to differences in auditory assessment methods and thresholds, follow-up durations, chemotherapy usage, and patient compositions. International guidelines and radiation dosimetric studies have recommended constraining the cochlear mean dose to less than 44–50 Gy, but the risk of SNHL remains high despite adherence to these constraints. Potential strategies to improve hearing outcomes in NPC survivors include cautious de-escalation of radiotherapy dose and volume, individualization of cochlear constraints, optimization of radiotherapy planning techniques, and the use of substitutes or alternative schedules for cisplatin-based chemotherapy. The addition of immune checkpoint inhibitors to chemoradiotherapy did not impact ototoxicity. Prospective studies that employ both objective and patient-reported auditory outcomes are warranted to test the long-term benefits of various approaches. This article aims to provide a comprehensive review of the incidence and radiation dose–toxicity relationship of SNHL in NPC survivors and to summarize potential strategies to optimize hearing outcomes in relation to nuances in radiotherapy planning and the selection of systemic therapy.

## 1. Introduction

Modern radiotherapy techniques, such as intensity-modulated radiation therapy (IMRT), have significantly improved the treatment outcomes of localized nasopharyngeal carcinoma (NPC) by enhancing disease control and reducing toxicity [1]. However, due to the proximity of the auditory apparatus to the nasopharynx, sensorineural hearing loss (SNHL) remains a major late complication in NPC survivors. Radiation-induced SNHL arises from direct damage to non-regenerating cochlear hair cells, as well as an indirect radiation-induced bystander effect triggered by oxidative stress and the inflammatory response [2]. The higher frequency hearing range is particularly susceptible to radiation-induced damage due to the low levels of the antioxidant glutathione in the basal outer hair cells, which are responsible for processing high-frequency sounds [3].

The incorporation of cisplatin as part of the definitive treatment regimens for NPC also contributes to ototoxicity. Cisplatin-induced ototoxicity is primarily driven by mitochondrial and DNA damage to the outer hair cells, spiral ganglion neurons, and stria vascularis of the cochlea, as well as the generation of reactive oxygen species that leads to inflammation and apoptotic cell death [4,5]. Importantly, these detrimental effects to the cochlea occur in a dose-dependent manner. Along with the increasing use of contemporary cisplatin-based induction or adjuvant therapy in locoregionally advanced NPC, the risk of ototoxicity in NPC survivors is further exacerbated.

**Figure 1:**
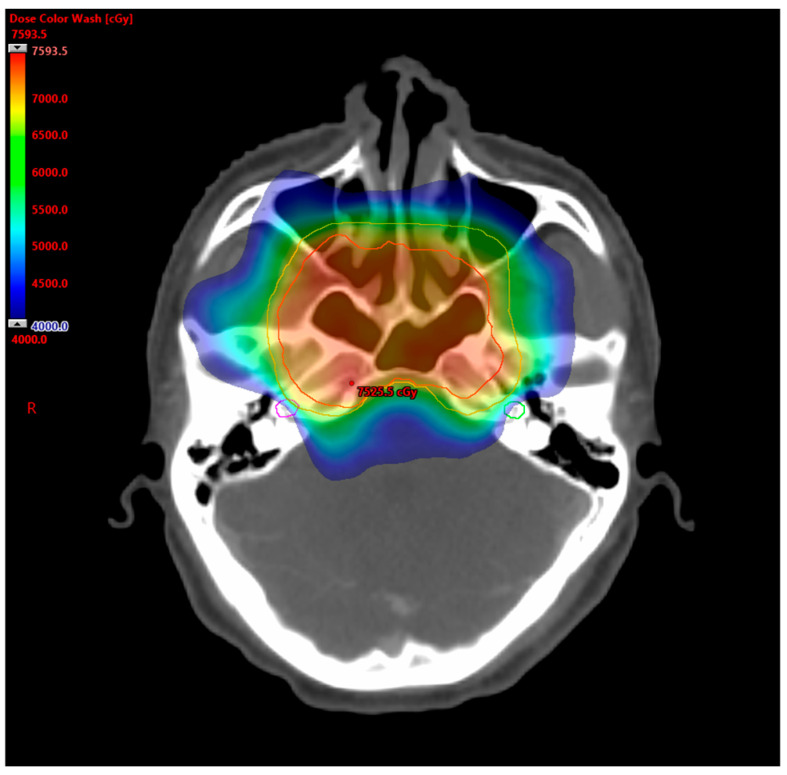
Cochlear-sparing IMRT for NPC. This was a VMAT plan for a patient with T3N2M0 NPC. The radiation dose color wash was set to depict 40–75.9 Gy. The following structures are delineated: PTV70 (red line), PTV60 (orange line), right cochlea (purple line), and left cochlea (green line). The mean doses of right and left cochlea were 44.8 Gy and 39.8 Gy, respectively.

During the era of conventional 2-dimensional (2D) radiotherapy for NPC, the reported incidence of SNHL was substantially high, ranging from 24% to 54% [6,7,8,9]. The advent of IMRT has enabled the delivery of highly conformal radiation to the planning target volume, thereby lowering the incidence of SNHL by better sparing the auditory apparatus (Figure 1). Over the past decade, there has been a growing body of literature on the dose–toxicity relationship of radiation-induced cochlear damage, allowing for the practical application of appropriate constraints during radiotherapy planning. Hearing outcomes may also be improved through cautious de-escalation in radiotherapy dose and volume, along with careful considerations of dosage, scheduling, and potential substitutes for cisplatin as a radiosensitizer.

This review aims to summarize the incidence and radiation dose–toxicity relationship of SNHL in NPC survivors treated with modern radiotherapy and systemic therapy. Potential strategies for optimizing hearing outcomes in relation to radiotherapy planning and the selection of systemic therapy are also discussed.

## 2. Incidence of SNHL after IMRT for NPC

Several observational studies have reported the incidence of SNHL after IMRT for NPC, most of which had a retrospective design (Table 1) [10,11,12,13,14,15,16,17,18,19,20,21,22,23,24]. The median age of the patients was approximately 50 years, although two studies specifically recruited younger populations, with one enrolling patients under 20 years of age [18] and the other under 30 years of age [24]. Cisplatin was used as a concurrent radiosensitizer in most studies. The mean follow-up period varied significantly from 1.3 years to 9.9 years.

Across the studies conducted in the era of IMRT, the reported incidence of SNHL ranged from 1.0% to 46.0% [10,11,12,13,14,15,16,17,18,19,20,21,22,23,24]. Heterogeneous assessment methods and thresholds have been used to define SNHL. Table 2 shows the various definitions of hearing impairment outlined in commonly employed grading scales [25,26,27]. Most studies utilized either pure tone audiometry (PTA) or the Common Terminology Criteria for Adverse Events (CTCAE) to define SNHL. For PTA, hearing impairment was defined as an increase in the bone conduction threshold (BCT) at low frequencies (0.5–2 kHz) or high frequency (4 kHz) compared to baseline values. CTCAE, on the other hand, allows for both audiometry-based assessments and physicians’ grading of limitations in activities of daily living (ADL) or the need for therapeutic intervention.

In addition to these physician-based assessment tools, there appears to be an emerging trend toward subjective assessment to define hearing impairment. For instance, in a recent study [24], post-radiation hearing impairment was assessed using the Hearing Handicap Inventory for Adult (HHIA)-Screening version, a self-administered questionnaire. This study found that nearly 30% of NPC survivors experienced self-perceived severe hearing impairment after IMRT. HHIA has been validated to correlate with pure tone sensitivity [28] and has been demonstrated to be a more robust indicator of social and emotional well-being when compared to the measured hearing loss [29]. Integrating patient-reported outcomes into the evaluation of hearing loss assessment may facilitate the development of management strategies that are targeted to clinically meaningful endpoints.

Across the observational studies, the incidence of PTA-defined SNHL among post-IMRT NPC survivors ranged from 7.7% to 46.0%, which appeared to be higher than those defined by CTCAE (7.0–27.5% for G2, 1.7–17.2% for ≥ G3). There are several possible explanations for this numerical difference. First, the PTA-defined SNHL incidence was typically reported on a per-ear basis, whereas the CTCAE-defined incidence was reported on a per-patient basis, which may have counted bilateral hearing loss as a single event. In addition, PTA-defined SNHL focuses on specific hearing frequencies, which may not fully correlate with a patient’s ADL. For example, all studies that used PTA included high-frequency hearing loss at 4 kHz, as it is known to be more susceptible to the detrimental effects of radiation [3,30]. However, this measurement may not accurately reflect the impact on patients’ ADL, as the normal speech range occurs at a lower frequency range of 0.5–2 kHz [7].

It is worth noting that the reported risk of SNHL could also be influenced by the concurrent use of cisplatin, which was not accounted for by all studies. For instance, a study by Wang et al. demonstrated a significantly higher incidence of SNHL in patients who received a cumulative cisplatin dose exceeding 200 mg/m^2^ [15]. Over the recent decade, there has been a paradigm shift toward the use of extended chemotherapy in locoregionally advanced NPC. Both the ASCO and ESMO guidelines have recommended the adoption of either induction or adjuvant chemotherapy in addition to standard chemoradiotherapy [31,32]. These approaches, albeit effective, are associated with high cumulative cisplatin doses of up to 480 mg/m^2^ to 540 mg/m^2^. Given the heightened risk of compounded ototoxicity, more stringent radiation dose constraints for the cochlea may be required to adequately protect NPC patients from SNHL.

Another important consideration when examining the incidence of SNHL is the duration of follow-up. Early-onset hearing impairment is common among NPC patients who undergo definitive radiotherapy, but it may be unrelated to cochlear injury. Early prospective studies during the conventional 2D RT era indicated that a substantial proportion of patients developed otitis media as an acute adverse effect of irradiation, which is typically transient and resolves spontaneously within two years [7,8]. This finding is supported by modern IMRT cohorts, which also showed that radiation to the cochlea and inner ear was associated with late-onset high-frequency SNHL (24 months after RT completion) but not early-onset SNHL (within 12 months of RT completion) [22]. Considering this latency period, long-term follow-up for at least two years is necessary to accurately determine the incidence of radiation-induced SNHL. Notably, two studies [23,24] had an extended follow-up period of more than 8 years, reporting incidences of hearing impairment as high as 40%. In the absence of non-irradiated controls, their interpretations must be taken with caution, as age-related hearing loss and patients lost to follow-up may confound the incidence of SNHL.

## 3. Radiation Dose–Toxicity Relationship of SNHL

The radiation dose to several components of the auditory apparatus may be relevant to radiation-induced SNHL. A contouring atlas was developed to promote standardized delineation of organs at risk in patients with NPC receiving IMRT [33]. It was recommended that four auditory sub-structures should be contoured, namely, the internal auditory canal (IAC), cochlea, tympanic cavity, and bony part of the Eustachian tube. Their boundaries should be delineated on the bone window of simulation computer tomography images.

Despite the meticulous auditory OARs proposed for NPC radiotherapy planning, most studies have focused on the cochlea when evaluating the dose–response relationship of radiation ototoxicity. Given the small size of the cochlea (approximately 0.2 mL), it is reasonable that mean dose was the most commonly investigated parameter for establishing constraints.

Table 3 summarizes the studies on auditory constraints proposed for NPC radiotherapy planning and the corresponding incidence of SNHL stratified according to these constraints [11,15,20,21,22,24,34,35,36,37]. The proposed cochlear constraints were consistently set in the range of Dmean <44–50 Gy. These proposals are in line with the recommendations from QUANTEC and current international guidelines [38,39], which suggest limiting the cochlear Dmean to under 45 Gy. Understanding that this target may not be realistically achievable in locally advanced NPC, guidelines have also set a second constraint of cochlear Dmean <55 Gy for radiotherapy planning [39].

Other than the cochlea, few studies have suggested the use of the IAC as a key auditory OAR. Two studies proposed IAC constraints of 32.7 Gy and 50 Gy, below which the SNHL rates were <21.6% and <25.8%, respectively [11,20]. In another study with a longer follow-up period, IAC V50 <40% and V60 <40% were recommended as dose constraints to limit self-reported hearing impairment. Two nomograms have been constructed using these metrics to assist with probability prediction in radiotherapy planning [24].

Instead of using a single constraint to dichotomize the risk of SNHL, studies have attempted to establish a quantitative relationship between cochlear dose and hearing loss in NPC survivors. In a recent retrospective study, Yip et al. estimated that each 10 Gy increase in cochlear Dmean would result in a 5 dB increase in BCT at 4 kHz [22]. Similarly, Peuker et al. developed a normal tissue complication probability model for the inner ear, estimating predicted toxicity rates of 25% and 50% at mean doses of 44 Gy and 65 Gy, respectively [37]. These findings highlight the merit of minimizing cochlear dose beyond the standard constraints to further reduce the risk of permanent hearing loss.

## 4. Prevention Strategy

### 4.1. De-Escalating Radiotherapy

In the era of IMRT, standard radiation doses of 70 Gy have demonstrated high local control rates of over 80–90% [40,41]. In radiotherapy planning, the clinical target volume (CTV), encompassing the gross tumor volume (GTV) along with a margin for potential subclinical disease extension, is of utmost significance as it is the volume that must be adequately treated to achieve a cure. The international guidelines on CTV delineation for NPC proposed by Lee et al. [42] recommend including the entire nasopharynx within either the full therapeutic volume (70 Gy) or prophylactic volume (lower dose), rather than relying on geometric expansion from the gross tumor. In addition, the petrous apex is routinely included within the prophylactic volume regardless of the local tumor extent. This blanket target delineation approach, when indiscriminately applied to all primary tumors, may result in unnecessarily high radiation doses to the auditory apparatus, particularly in patients with early lateralized diseases. Alternative target delineation methods to individualize the primary CTV according to T-stage and tumor topography have been proposed (Table 4) [43,44,45,46,47,48,49,50,51,52,53,54,55,56,57,58,59,60].

The local extension of NPC typically follows a stepwise spread pattern, with over 70% of primary tumors exhibiting asymmetrical infiltration into the skull base structures [61]. This raises the potential of sparing the contralateral organs at risk in lateralized tumors. In a retrospective study conducted by Xie et al. focusing on unilateral NPC (defined as tumors not crossing the midline according to endoscopy and MRI), the primary CTV was delineated using 1.5–2 cm geometric expansion from the GTV without prophylactic inclusion of the contralateral skull base structures [43]. After a median follow-up of 7 years, no out-of-field local recurrence was observed in the contralateral unirradiated structures. Importantly, this approach resulted in a low average Dmean of the contralateral middle ear of 24.1 Gy. No high-grade acute hearing loss was observed, and only 1 out of 95 contralateral ears developed late hearing impairment.

A more recent study by Wang et al. proposed an individualized method for delineating the CTV that combines geometric expansion and the consideration of anatomical structures [45]. This approach involves the division of the adjacent structures of the nasopharynx into different tiers, taking into account the pattern of tumor spread. The CTV was then delineated using a combination of geometric expansion and the inclusion of at-risk structures based on the extent of the primary tumor. Specifically, only the ipsilateral petrous apex was included in primary CTV. With a median follow-up period of 66.6 months, both the 5-year local recurrence-free survival and overall survival (OS) rates exceeded 90%, and the incidence of grade 3 hearing impairment was remarkably low (0.2%).

Another potential strategy is response-adaptive de-escalation of radiotherapy. The use of induction chemotherapy before concurrent chemoradiotherapy has shown significant improvements in OS in patients with advanced NPC [62]. However, the optimal CTV following response to induction chemotherapy remains controversial. Current international guidelines suggest that the pre-induction chemotherapy volume should ideally receive the full therapeutic dose regardless of any shrinkage that may occur after induction chemotherapy [42]. In a randomized controlled trial, Xiang et al. [44] tested the feasibility of radiation volume de-escalation, with the post-induction chemotherapy volume receiving the full therapeutic dose and the pre-induction chemotherapy volume treated with a lower prophylactic dose. Compared with the control group, in which patients received a full therapeutic dose to the pre-induction chemotherapy volume, patients who underwent de-escalated radiotherapy had similar 5-year locoregional relapse-free survival. While differences in cochlear dose have not been reported, patients in the reduced-volume group had a significantly lower incidence of late hearing loss (52.2% vs. 69.1%), suggesting potential merits in preserving auditory function with this de-escalation approach.

In addition to restricting high-dose radiation to the post-induction chemotherapy tumor volume, several studies have proposed dose de-escalation in patients who exhibit tumor responses. In a retrospective, propensity score-matched cohort analysis conducted by Yao et al. [46], the impact of reducing the radiation dose to the high-risk CTV was evaluated in adolescent patients with locally advanced NPC who achieved complete or partial response after two to four cycles of induction chemotherapy. Compared with patients who received standard doses (66–70 Gy), patients in the reduced-dose (60–65.9 Gy) group had similar survival outcomes but a lower incidence of severe late toxicity, including grade 3–4 hearing loss (10.6% vs. 5.9%). This response-adaptive approach was tested in a phase II single-arm prospective trial by Luo et al. [47]. The trial stratified patients based on their response to induction chemotherapy: for patients whose tumors achieved complete response (CR) or partial response (PR), a lower dose of 60 Gy was given to the GTV, 54 Gy to the high-risk CTV, and 48 Gy to the low-risk CTV, whereas standard doses of 70 Gy, 60 Gy, and 54 Gy were given for static or progressed tumors. This approach resulted in excellent 3-year progression-free survival (PFS) and OS rates of 91% and 100%, respectively. The incidence of grade 1–2 hearing impairment was low at 14%, and no high-grade event was reported.

Apart from using tumor responses to stratify patients for treatment de-escalation, there is also growing interest in individualizing radiation doses based on biomarker responses. Guo et al. investigated the role of de-escalating the radiation dose in low-risk stage III NPC with low pre-treatment plasma EBV DNA levels [48]. After two cycles of induction chemotherapy, patients who achieved tumor responses and had undetectable plasma EBV DNA received 60–66 Gy, whereas those who did not meet these criteria were treated with 70 Gy. This approach resulted in an encouraging 3-year PFS rate of 88.8%. Importantly, the incidence of grade 1–2 hearing impairment in patients treated with a reduced dose was low at 14.7%, in contrast to 41.4% in patients who received standard radiotherapy dose.

Ultimately, given the prevailing importance of local tumor control over the risk of radiation-induced SNHL, de-escalation in radiotherapy volume or dose should be carefully approached with individualized considerations of the anatomic relationships between the tumor and cochlea, baseline auditory function, and patient preference. Prospective clinical trials that incorporate auditory outcomes as secondary endpoints will help to ensure safety and provide valuable insight into the magnitude of the benefit.

### 4.2. Individualizing Cochlea Dose Constraints

In the current international dose prioritization guidelines for NPC, each OAR was assigned a “goal” constraint and a “maximum acceptance” constraint [39]. The Dmean constraints for the cochlea were set to ≤45 Gy and ≤55 Gy, respectively. Among the list of OARs, the cochlea was assigned the lowest dosimetric priority (priority 4).

Instead of using a one-size-fits-all constraint on the cochlea, a risk-stratified approach has been proposed to achieve better cochlea sparing across different T stages. In a dosimetric study by Zhang et al. using step-and-shoot IMRT, various strategies to reduce cochlear dose were attempted [49]. These included setting tiered cochlear constraints, upgrading the protection priority for the cochlea, and modification of the radiation beam angle. For T1 tumors, the maximal cochlear dose was set to <45 Gy. For T2 or T3 tumors of which the PTV overlapped with the cochlea, a constraint of V50 < 50% was applied. For T3 or T4 tumors where the PTV encircled the cochlea, the maximum cochlear dose was limited to <103% of the prescription dose. In addition, during plan optimization, the dosimetric priority of the cochlea was upgraded to the same level as the salivary glands. Compared to the control plans, the above approach achieved a significantly lower cochlear Dmean without compromising target coverage parameters or other important OARs. The average ipsilateral and contralateral cochlear Dmeans were reduced from 50.6 Gy to 46.2 Gy and 50.0 Gy to 43.9 Gy, respectively.

### 4.3. Selection of Radiotherapy Techniques

Compared with standard step-and-shoot IMRT, volumetric modulated arc therapy (VMAT) may achieve superior dose homogeneity and lower doses to the OARs through modulation of the multileaf collimator (MLC) pattern, as well as gantry rotation speed and dose rate [63]. In the context of NPC, several investigators have examined the benefit of VMAT on cochlear dose [50,51,52,53]. In a planning study that compared the dosimetric outcomes between VMAT and step-and-shoot IMRT, jaw tracking was employed in VMAT plans to ensure the cochlea was kept outside the treatment field during gantry rotation, thereby reducing the interleaf leakage from MLC [52]. This technique resulted in significantly lower average cochlear Dmeans (right: 18.5 Gy vs. 26.1 Gy; left: 15.0 Gy vs. 24.1 Gy). In another large retrospective study that included 627 NPC patients, the use of VMAT also resulted in significantly lower rates of grade 1–2 (16.4% vs. 25.1%) and grade 3–4 (0.9% vs. 3.1%) ototoxicity than step-and-shoot IMRT, suggesting that VMAT may be the preferred radiotherapy planning technique for NPC [51].

The use of proton therapy in the treatment of NPC has gained attention due to its dosimetric advantages in minimizing the dose to normal structures, which are particularly important when the primary tumor and OARs are in close proximity. Several studies have reported the auditory outcomes of proton therapy for NPC [54,55,56]. In cohorts that utilized modern pencil-beam proton radiotherapy, the reported cochlear Dmean ranged from 31 Gy to 42 Gy. No high-grade hearing impairment was reported, with a low incidence of grade 2 events of 4–7% at 2 years after treatment [54,55]. In another retrospective dosimetric study, Anderson et al. showed that proton therapy could achieve a significantly lower cochlear Dmean (25.5–33.2 Gy) when compared to treatment with photon-based IMRT (31.5–41.8 Gy) [56]. These data support clear dosimetric advantages of proton therapy on the cochlea in NPC radiotherapy planning. Prospective studies that compare with proton- and photon-based IMRT are warranted to quantify the magnitude of the benefits in auditory outcomes.

### 4.4. De-Escalating Systemic Therapy

Since the risk of SNHL is compounded by the use of cisplatin, de-escalating systemic therapy in carefully selected patient subgroups may also reduce the risk of SNHL in patients with localized NPC.

For stage II NPC, recommendations from international guidelines suggest that concurrent chemoradiotherapy with cisplatin may be considered in selected patients [31,32,64,65], based on the survival benefit shown in a randomized clinical trial that used 2DRT [66]. However, in the era of IMRT, increasing evidence suggests that radiotherapy alone can achieve excellent clinical outcomes [67,68,69]. In a recent randomized controlled trial, Tang et al. [57] examined the feasibility of omitting concurrent cisplatin in low-risk NPC, defined as stage II or T3N0M0 (AJCC 7th edition) without adverse features, namely, lymph nodes larger than 3 cm, level IV, or VB nodal involvement, extracapsular extension, and plasma EBV DNA levels higher than 4000 copies/mL. With a median follow-up of 46 months, radiotherapy alone has led to survival outcomes that are non-inferior to those of chemoradiotherapy. The omission of concurrent cisplatin appeared to have a small benefit in reducing the risk of hearing impairment (grades 1–2, 40% vs. 47%).

Treatment with fewer concurrent cisplatin cycles has also been investigated. Traditionally, concurrent cisplatin in chemoradiotherapy for NPC consists of 3 tri-weekly cycles at a dose of 100 mg/m^2^ [40,70]. In a phase II randomized non-inferiority trial, Li et al. [58] compared 2 cycles versus 3 cycles of concurrent cisplatin in stage III to IVB NPC patients with plasma EBV DNA <4000 copies/mL. While the 3-year survival outcomes were similar between the two groups, patients who underwent 2 cycles of concurrent cisplatin had a lower incidence of hearing impairment of any grade (22.9% vs. 35.2%) and a trend toward lower severe hearing impairment (0.6% vs. 3.6%), suggesting that 200 mg/m^2^ of cisplatin may be a sufficient dose for low-risk loco-regionally advanced diseases.

Traditionally, fractionated cisplatin delivery schedules, such as the weekly regimen, have been thought to be less ototoxic [71]. A recent randomized controlled trial compared 2 cycles of concurrent once-every-3-weeks cisplatin (100 mg/m^2^) to 6 cycles of weekly cisplatin (40 mg/m^2^) in NPC patients [72]. Surprisingly, while the 3-year failure-free survival rates were similar between the two groups, the rate of late grade 3–4 hearing impairment was significantly lower in patients treated with once-every-3-weeks cisplatin (9.6% vs. 16.5%). This finding may be attributable to the higher cumulative cisplatin dose in the weekly than the once-every-3-weeks group (median, 220 mg/m^2^ vs. 200 mg/m^2^), further supporting the clinical merit of safe de-escalation of cisplatin dose in the treatment for NPC.

In search of alternative concurrent agents to cisplatin, researchers have developed platinum derivatives to mitigate the toxicity associated with cisplatin. Tang et al. showed that nedaplatin-based concurrent chemoradiotherapy is non-inferior to standard cisplatin-based concurrent chemoradiotherapy in terms of 2-year PFS, with a significantly lower rate of any grade (14% vs. 23%) and grade 3 hearing impairment (2% vs. 6%) [59]. On the other hand, Lv et al. [60] showed that S-1, a 5-fluorouracil prodrug, can be used as a concurrent agent with IMRT in NPC treatment. Its combination with IMRT resulted in a favorable 3-year PFS of 87.4%, with grade 1 hearing impairment observed in only 2.3% of patients, without grade 2 or above events.

A substantial body of research is currently exploring the integration of immune checkpoint inhibitors with radiotherapy for NPC. A recent randomized phase III trial has indicated that the addition of sintilimab, a PD-1 inhibitor, to standard induction-concurrent chemoradiotherapy significantly improved event-free survival in patients with loco-regionally advanced disease [73]. Importantly, this intensification of treatment did not adversely affect auditory outcomes, as evidenced by the comparable rates of all-grade hearing impairment between the sintilimab group and the standard therapy group (35% vs. 34%). Additionally, ongoing clinical trials are investigating the efficacy of adjuvant immune checkpoint inhibitors following standard chemoradiotherapy (NCT03544099, NCT04910347). Should these approaches demonstrate non-inferiority relative to standard induction or adjuvant chemotherapy, they may help to reduce the cumulative cisplatin dose from extended chemotherapy cycles, thereby reducing the risk of SNHL in patients with advanced NPC. This approach may hold particular relevance for those high-risk patients who might otherwise be undertreated without the use of induction or adjuvant cisplatin-based chemotherapy.

## 5. Management

Radiation- or cisplatin-induced SNHL is an irreversible complication for which no standard treatment is currently available. Among the available options, hearing aids offer a non-surgical and accessible treatment for patients with serviceable hearing [74]. For patients with severe SNHL, sound amplification by hearing aids would be ineffective, and cochlear implantation may be considered.

Cochlear implants transduce acoustic energy into electrical signals that stimulate the surviving spiral ganglion cells of the auditory nerve [75]. Although whether the retro-cochlear auditory pathway is damaged following chemoradiotherapy remains controversial [76,77], retrospective studies and case series have demonstrated the efficacy and safety of cochlear implants in terms of improving hearing and speech performance in NPC survivors with severe SNHL [77,78,79]. Co-existing radiation-induced complications, such as chronic suppurative otitis media (CSOM) and temporal bone osteonecrosis, can complicate the candidacy for cochlear implants. In these situations, concurrent surgeries including mastoidectomy, temporal bone resection, and external auditory canal elimination may be required [79,80]. The bone-anchored implantable hearing aid system may represent a potential solution for post-irradiation patients with CSOM, recurrent ear discharge, and osteoradionecrotic bony ear canal ulcers. The system utilizes an osseointegrated titanium implant to directly transmit sound to the functioning cochlea, bypassing the external and middle ear mechanism. A prospective longitudinal study showed that NPC patients had improved subjective hearing clarity and reduced otorrhea rate with this system [81]. These surgical options may be particularly beneficial for those high-risk NPC patients who are not eligible for treatment de-escalation.

Emerging therapies, such as signal pathway inhibitors, antioxidant drugs, and gene therapy, are currently under investigation as potential treatment options, although none are currently approved for the treatment of SNHL in NPC survivors [2]. For example, AM-111, a c-Jun N-terminal kinase (JNK) inhibitor, was found to significantly improve hearing outcomes in patients with profound idiopathic sudden SNHL [82]; it is expected to be useful in radiation-induced SNHL, which also involves the JNK signaling pathway in its pathogenesis [83]. As radiation-induced SNHL is closely associated with oxidative stress and the inflammatory response, antioxidant drugs appear to be reasonable agents in treatment. Many antioxidants, such as melatonin and amifostine, have been tested in animals but not humans. Methylprednisolone use during RT has been shown to reduce SNHL one year after RT in a small prospective trial, but larger studies with longer follow-up are needed [84]. On the other hand, cochlear hair cell regeneration using induced pluripotent stem cells and epigenetic drugs has emerged as a promising treatment option, although many of them are still in their preclinical phases of drug development [85].

## 6. Conclusions

SNHL is a debilitating and irreversible late complication in NPC survivors. The reviewed studies reported a wide range of SNHL incidence following contemporary radiotherapy and systemic treatments for NPC, which can be attributed to the heterogeneity in assessment methods, auditory endpoints, cumulative cisplatin dose, and follow-up durations. There is an emerging trend toward incorporating patient-reported outcomes to evaluate hearing impairment, highlighting the importance of a comprehensive approach that encompasses both objective and subjective measures.

In terms of prevention, constraining the cochlear Dmean to under 44–50 Gy may reduce radiation ototoxicity, but the incidence of SNHL remained relatively high even when these constraints were met. Other potential strategies to mitigate the risk of SNHL in NPC survivors include response-adaptive de-escalation of radiotherapy volume and dose, individualization of cochlear constraints according to tumor stage, employing specific radiotherapy planning techniques, and cautious de-escalation of systemic therapy. Prospective studies are warranted to evaluate the long-term outcomes of these approaches.

## Figures and Tables

**Table 1:** Summary of studies reporting incidence of SNHL in NPC patients who underwent IMRT.

Study	Study Design	*n*	Median Age (Years)	Median FU (Months)	Cisplatin Use	Cisplatin Dose	Definition of SNHL	Incidence
Hsin et al., 2010 [10]	Prospective cohort	26 ^a^	Mean 43	36	88.5%	Concurrent 30 mg/m^2^ weekly	Increase in BCT ≥20 dB at 4 kHz	46%
Petsuksiri et al., 2011 [11]	Retrospective cohort	27 ^a^	47.5 ^b^	27.5 ^b^	97% ^b^	Concurrent 100 mg/m^2^ Q3w Adjuvant 80 mg/m^2^ with 5FU Q3w	Increase in BCT of ≥15 dB at 4 kHz	37% ^c^
Leung et al., 2013 [12]	Prospective cohort	72	46.5	41	62.5%	NR	As per RTOG	3% (G3)
Lee et al., 2014 [13]	Retrospective cohort	444 ^a^	Mean 52	81.6 ^b^	87%	NR	CTCAE ≥G3	17.2%
Ou et al., 2015 [14]	Retrospective cohort	869	Mean, 49	54.3	84.8%	Induction or adjuvant 75 mg/m^2^ with docetaxel and/or 5FU and/or gemcitabine Q3w for 2–3 (induction) or Q4w for 2–3 cycles (adjuvant) Concurrent 80 mg/m^2^ Q3w or 40 mg/m^2^ weekly	CTCAE	13.0% (any grade) 1.7% (≥G3)
Wang et al., 2015 [15]	Retrospective cohort	51	42	60	100%	Induction 80 mg/m^2^ with 5FU Q3w for 2–3 cycles Concurrent 80 mg/m^2^ Q3w or 40 mg/m^2^ weekly Adjuvant 80 mg/m^2^ with 5FU Q3w for 3–4 cycles	Increase in threshold ≥15 dB	At 0.5–2 kHz: 12.7% At 4 kHz: 42.2% ^c^
Zheng et al., 2015 [16]	Prospective cohort	208	42	78	38.9%	NR	As per LENT/SOMA	47.1% (G1) 19.7% (G2) 0.96% (G3)
Huang et al., 2016 [17]	Cross-sectional study	100 ^a^	46.2	≥60	73%	NR	CTCAE ≥G2	26.0%
Qiu et al., 2017 [18]	Retrospective cohort	102 ^a^	Range, 7–20 ^b^	52 ^b^	97.1%	Induction with 5FU Q3w for 1–3 cycles Concurrent 30 mg/m^2^ weekly or 80–100 mg/m^2^ Q3w	CTCAE ≥G2	22.5%
Chan et al., 2018 [19]	Prospective and retrospective cohort	142	56.8 (RT) 49.6 (CRT) 52.5 (iCRT)	14.7 (RT) 15.2 (CRT) 17.8 (iCRT)	78.2%	Induction 75 mg/m^2^ Q3w for 3 cycles Concurrent 40 mg/m^2^ weekly	CTCAE ≥G2	27.5%
Zhu et al., 2019 [20]	Retrospective cohort	70	44	69	NR	70 mg/m^2^ with 5FU Q3w	Increase in hearing threshold ≥15 dB	At 0.5–2 kHz: 7.69% At 4 kHz: 35.9% ^c^
Inada et al., 2022 [21]	Prospective cohort	74	55	50	100%	Concurrent 80 mg/m^2^ Q3w	CTCAE ≥G2	5-year incidence 26%
Yip et al., 2022 [22]	Retrospective cohort	81	53.5 (RT) 48.9 (CRT) 49.1 (iCRT)	38	64.0%	Induction 100 mg/m^2^ Q3w for 3 cycles Concurrent 100 mg/m^2^ Q3w or 40 mg/m^2^ weekly	Increase in BCT ≥15 dB within 12 months after completion of RT (early) and at least 2 years after completion of RT (late)	At 4 kHz, Early: 19.9% Late: 32.2%
Doi et al., 2023 [23]	Retrospective cohort	43	55	119	100%	Concurrent 80 mg/m^2^ for 2–3 cycles Adjuvant 70 mg/m^2^ with 5FU for 2–3 cycles	CTCAE ≥G2	7%
Chen et al., 2024 [24]	Retrospective cohort	588	27	103.4	84.8%	Concurrent weekly or Q3w regimen Median 160 mg/m^2^	The Hearing Handicap Inventory for Adult-Screening version (HHIA-S)	Hearing impairment: 39.5% Severe hearing impairment: 29.7%

Abbreviations: 5FU, 5-fluorouracil; BCT, bone conduction threshold; CRT, concurrent chemoradiotherapy; CTCAE, Common Terminology Criteria for Adverse Events; iCRT, induction-concurrent chemoradiotherapy; LENT/SOMA, Late Effects Normal Tissue Task Force (LENT)-Subjective, Objective, Management, Analytic (SOMA) scales; RT, radiotherapy; NR, not reported; Q3w, every three weeks; Q4w, every four weeks; SNHL, sensorineural hearing loss. ^a^ For patients treated with IMRT in the whole cohort. ^b^ For patients in the whole cohort. ^c^ Incidence per ear.

**Table 2:** Ototoxicity grading scale for hearing impairment.

	Grade 1	Grade 2	Grade 3	Grade 4
CTCAE v4.0/v5.0 ^a^ [25] Enrolled in a monitoring program (1, 2, 3, 4, 6, and 8 kHz audiogram)	Threshold shift of 15–25 dB averaged at 2 contiguous test frequencies in at least one ear	Threshold shift of >25 dB averaged at 2 contiguous test frequencies in at least one ear	Threshold shift of >25 dB averaged at 3 contiguous test frequencies in at least one ear; therapeutic intervention indicated	Profound bilateral hearing loss (>80 dB at 2 kHz and above)
CTCAE v4.0/v5.0 [25] Not enrolled in monitoring program	Subjective change in hearing in the absence of documented hearing loss	Hearing loss but hearing aid or intervention not indicated; limiting instrumental ADL	Hearing loss with hearing aid or intervention indicated; limiting self-care ADL	Non-serviceable hearing
LENT/SOMA [26]	<10 dB loss in one or more frequencies; minor loss, no change in daily activities	10–15 dB loss in one or more frequencies; frequent difficulties with faint speech	>15–20 dB loss in one or more frequencies; frequent difficult with loud speech; hearing aid required	>20 dB loss in one or more frequencies; complete deafness
RTOG/EORTC Toxicity Criteria [27]	Mild	Moderate	Severe	Life-threatening or disabling

Abbreviations: ADL, activities of daily living; CTCAE, Common Terminology Criteria for Adverse Events; LENT/SOMA, Late Effects Normal Tissue Task Force—Subjective, Objective, Management, Analytic; RTOG/EORTC, the Radiation Therapy Oncology Group and the European Organization for Research and Treatment of Cancer. ^a^ CTCAE v3.0, used in some studies, has similar definitions with the exception of defining the threshold shift to be 25–90 dB in grades 2 and 3 and >90 dB in grade 4.

**Table 3:** Summary of studies on dosimetric constraints of the auditory apparatus in radiotherapy planning for NPC.

Study	*n*	RT Technique	Proposed Parameter	Proposed Dosimetric Constraint	Adjusted for Cisplatin?	Adjusted for T Staging?	Median FU (Months)	Incidence of SNHL Per Ear (Low Risk Group vs. High Risk Group with Reference to the Proposed Dosimetric Cut-Off Point)
Chen et al., 2006 [34]	22	IMRT 68% 3DCRT 32%	Dmean cochlea	≤48 Gy	No	No	29	24% vs. 61% at 4 kHz
Chan et al., 2009 [35]	87	IMRT 69% 3DCRT 31%	Dmean cochlea	<47 Gy	Yes	No	24	14% vs. 31%
Petsuksiri et al., 2011 [11]	68	IMRT 40% 2DRT 60%	Dmean IAC	≤50 Gy	Yes	No	27.5	25.81% vs. 52.17%
Wei et al., 2014 [36]	72	IMRT 100%	Dmean cochlea	<46 Gy	No	No	60	NR
Wang et al., 2015 [15]	51	IMRT 100%	D0.1ml cochlea	<39.8 Gy	Yes	Yes	60	4.3% vs. 20.0%
Zhu et al., 2019 [20]	70	IMRT 100%	Dmax IAC	<42.13 Gy	No	Yes	69	19.4% vs. 41.9%
			Dmean IAC	<32.72 Gy				21.6% vs. 42.5%
Yip et al., 2022 [22]	81	IMRT 100%	Dmean cochlea	<40 Gy	Yes	No	38	0% vs. 29.4% Every 10 Gy increase in Dmean cochlea leads to 5 dB increase in BCT at 4 kHz
			Dmean inner ear	NR				Every 10 Gy increase in Dmean inner ear leads to 6 dB increase in BCT at 4 kHz
Inada et al., 2022 [21]	74	IMRT 100%	Dmean ipsilateral inner ear	<44 Gy	No	No	50	18% vs. 42%
Peuker et al., 2022 [37]	46	IMRT 97.8% 3DCRT 2.2%	Dmean inner ear	<44Gy	No	No	31.2	25% vs. NR ^a^
			Dmax inner ear	<58 Gy				25% vs. NR ^a^
Chen et al., 2024 [24]	588	IMRT 100%	HI:		Yes	No	103.4	NR
			V45 inner ear	<50%				
			V50 IAC	<40%				
			Severe HI:					
			Dmin IAC	<44 Gy				
			V60 IAC	<40%				

Abbreviations: 2DRT, conventional 2-dimensional radiotherapy; 3DCRT, 3-dimensional conformal radiotherapy; BCT, bone conduction threshold; Dmax, maximal dose; Dmean, mean dose; Dmin, minimal dose; FU, follow-up; HI, hearing impairment; IAC, internal auditory canal; IMRT; intensity-modulated radiotherapy; N, sample size; NR, not reported; RT, radiotherapy; SNHL, sensorineural hearing loss. ^a^ Incidence of inner ear toxicity (hearing impairment, tinnitus).

**Table 4:** Potential strategies to prevent SNHL in patients with NPC.

Prevention Strategy	Study	Strategy Details	*n*	Median FU (Months)	Auditory Outcomes
**De-escalating radiotherapy**					
Reducing treatment volume	Xie et al., 2022 [43]	CTV delineated by geometric expansion of gross tumor for unilateral NPC (defined as tumor confined to one side of NP and did not cross the midline by endoscopy and MRI) without including the whole nasopharyngeal mucosa	95	84	Low incidence of hearing impairment on tumor-contralateral side (1.1%)
	Xiang et al., 2023 [44]	GTV contoured according to post-induction chemotherapy tumor extent, with pre-induction chemotherapy tumor extent included in the high-risk prophylactic volume	233	98.4	Lower incidence of any grade hearing loss compared to control (52.2% vs. 69.1%)
	Wang et al., 2024 [45]	Divide the neighboring structures of nasopharynx into 4 levels; CTV delineated by geometric expansion of gross tumor plus the inclusion of neighboring structures according to the extent of primary tumor	1004	66.6	RTOG G1 hearing impairment 24.1%, G2 2.1%, G3 0.2%
Reducing radiation dose	Yao et al., 2023 [46]	Lower radiation dose to GTV (60–65.9 Gy) for patients with stage III–IVA (AJCC 8th edition) NPC who achieved tumor response after induction chemotherapy	132	75.2	Lower incidence of CTCAE G3–4 hearing loss compared to control (5.9% vs. 10.6%)
	Luo et al., 2023 [47]	Lower radiation dose to GTV (60 Gy) for patients with stage IVA–IVB (AJCC 7th edition) NPC who achieved tumor response after induction chemotherapy; those with SD or PD received the standard dose (70 Gy)	44	38.2	CTCAE G1–2 hearing impairment 14%, G3–4 0%
	Guo et al., 2023 [48]	Lower radiation dose to GTV (60 Gy) for patients with stage III (AJCC 8th edition) NPC who had pre-treatment plasma EBV DNA <4000 copies/mL and achieved tumor response and undetectable plasma EBV DNA after induction chemotherapy; a 6 Gy boost to residual tumors at the end of treatment	215	43.9	RTOG G1–2 hearing impairment 14.7% vs. 41.4%, G3 hearing impairment 0% vs. 1%
**Individualizing cochlea dose constraints**					
	Zhang et al., 2019 [49]	Based on T-stage, a cochlea-sparing plan was designed by individualizing dose constraints, and/or upgrading cochlea protection weight, and/or changing radiation beams angles	19	N/A	Significantly lower ipsilateral cochlear Dmean (46.2 Gy vs. 50.6 Gy) and contralateral cochlear Dmean (43.9 Gy vs. 49.9 Gy) compared to standard plans
**Radiotherapy techniques**					
Volumetric-modulated arc therapy	Gao et al., 2015 [50]	VMAT plan with the SmartArc planning algorithm in stage III or IV NPC	20	N/A	Significantly lower cochlear Dmean (42.7–43.8 Gy) than the control IMRT plan (47.6–47.8 Gy)
	He et al., 2020 [51]	VMAT plan in stage I–IVB NPC	627	≥12	Significantly lower cochlear Dmean in T3–4 tumor (45.08 Gy vs. 46.31 Gy) and lower ototoxicity (G1–2 16.4% vs. 25.1%, G3–4 0.9% vs. 3.1%) than the control IMRT plan
	Lamaj et al., 2021 [52]	VMAT with jaw tracking to ensure the cochlea would remain outside the treatment field whenever possible, or as as close to the cochlea as possible if very close to the PTV	20	N/A	Significantly lower cochlear Dmean (14.97–18.47 Gy vs. 24.09–26.05 Gy) than the control plan
	Nanda et al., 2023 [53]	VMAT plan in stage III or IV NPC	14	N/A	Significantly lower cochlear Dmean (38.05–38.26 Gy vs. 42.13–43.16 Gy) than the control IMRT plan
Proton therapy	Jiri et al., 2021 [54]	Three-field pencil-beam scanning IMPT with adaptive re-planning	40	24	RTOG G2 ototoxicity 7%, G3 or above 0%
	Williams et al., 2021 [55]	Two- to five-field pencil-beam scanning IMPT	26	25	CTCAE G1 hearing impairment 27%, G2 4%, ≥G3 0%
	Anderson et al., 2023 [56]	Pencil-beam scanning proton therapy	32	30	Significantly lower cochlear Dmean at the less spared side (33.2 Gy vs. 41.8 Gy) and better spared side (25.5 Gy vs. 31.5 Gy) than the control IMRT/VMAT plan
**De-escalating systemic therapy**					
Omission of concurrent cisplatin	Tang et al., 2022 [57]	Omit concurrent cisplatin in low-risk NPC, defined as stage II or T3N0M0 (AJCC 7th edition) without adverse features (all lymph nodes <3 cm, no level IV/VB involvement, no ECE, plasma EBV DNA level <4000 copies/mL)	341	46	RTOG G1–2 hearing impairment 40% vs. 47%, G3–4 1% vs. 1% compared to control group
Alternative cisplatin schedule	Li et al., 2022 [58]	Reduce cumulative cisplatin dose by using 2 cycles of concurrent cisplatin (100 mg/m^2^ every 3 weeks) instead of 3 cycles, in low-risk locally advanced NPC as defined by stages III to IVB (AJCC 7th edition) and plasma EBV DNA <4000 copies/mL	332	37.7	Lower rates of RTOG hearing impairment of any grade, 22.9% vs. 35.2%, and G3–4 hearing impairment, 0.6% vs. 3.6%
Alternative concurrent agent other than cisplatin	Tang et al., 2018 [59]	Use of nedaplatin as the concurrent agent with IMRT	402	48	Lower rates of RTOG hearing impairment of any grade, 14% vs. 23%, and G3, 2% vs. 6%
	Lv et al., 2019 [60]	Use of oral S-1 as the concurrent agent with IMRT in locally advanced NPC	131	24.5	RTOG G1 hearing impairment 2.3%, G2 or above 0%

Abbreviations: CTCAE, Common Terminology Criteria for Adverse Events; CTV, clinical target volume; Dmean, mean dose; EBV DNA, Epstein–Barr virus DNA; ECE, extracapsular extension; FU, follow-up; GTV, gross tumor volume; Gy: radiobiologically equivalent dose; IMPT: proton therapy with modulated intensity; PD, progressive disease; PTV, planning target volume; RTOG, Radiation Therapy Oncology Group (toxicity criteria); SD, stable disease.
